# Are Some Fungal Volatile Organic Compounds (VOCs) Mycotoxins?

**DOI:** 10.3390/toxins7093785

**Published:** 2015-09-22

**Authors:** Joan W. Bennett, Arati A. Inamdar

**Affiliations:** 1 Department of Plant Biology and Pathology, the State University of New Jersey, Rutgers University, New Brunswick, NJ 08901, USA; E-Mail: profmycogirl@yahoo.com; 2 The Genomics and Biomarkers Program, the John Theurer Cancer Center, Hackensack University Medical Center, Hackensack, NJ 07601, USA; E-Mail: inamdar@rci.rutgers.edu

**Keywords:** biogenic volatile organic compounds, fungi, mycotoxins, semiochemicals, toxins

## Abstract

Volatile organic compounds (VOCs) are carbon-compounds that easily evaporate at room temperature. Toxins are biologically produced poisons; mycotoxins are those toxins produced by microscopic fungi. All fungi emit blends of VOCs; the qualitative and quantitative composition of these volatile blends varies with the species of fungus and the environmental situation in which the fungus is grown. These fungal VOCs, produced as mixtures of alcohols, aldehydes, acids, ethers, esters, ketones, terpenes, thiols and their derivatives, are responsible for the characteristic moldy odors associated with damp indoor spaces. There is increasing experimental evidence that some of these VOCs have toxic properties. Laboratory tests in mammalian tissue culture and *Drosophila melanogaster* have shown that many single VOCs, as well as mixtures of VOCs emitted by growing fungi, have toxic effects. This paper describes the pros and cons of categorizing toxigenic fungal VOCs as mycotoxins, uses genomic data to expand on the definition of mycotoxin, and summarizes some of the linguistic and other conventions that can create barriers to communication between the scientists who study VOCs and those who study toxins. We propose that “volatoxin” might be a useful term to describe biogenic volatile compounds with toxigenic properties.

## 1. Introduction

In order to answer the deceptively simple question “Are some fungal organic volatile compounds (VOCs) mycotoxins?” it is necessary to define both “VOC” and “mycotoxin.” As we do so in the sections below, it becomes apparent that our “simple” question requires us to stand back and examine the way in which scientists use language and categorize the phenomena we study. Both fungal VOCs and mycotoxins are the products of fungal metabolism. Nevertheless, the set of definitions and circumscriptions surrounding fungal VOCs leads us to address a chemical class based on its physical properties (readily entering gas phase) while the set of definitions and circumscriptions concerning mycotoxins leads us to definitions based on physiological activity (toxicity). The large literature on volatile products of fungal metabolism is almost entirely devoted to their aroma and flavor properties, their use as indicators of fungal growth, or their activity as environmental signaling compounds (semiochemicals). Another large literature on mycotoxins focuses almost entirely on compounds that are biosynthesized via secondary metabolic pathways that have deleterious effects on humans and other vertebrates in low concentrations. Thus, we are dealing with “apples and oranges.” Or are we?

Currently, very little is known about the toxicity of most fungal VOCs, their biosynthetic origin, their genomic signatures, or the phylogenetic relationships of the enzymes involved in their production. What's more, the group of scientists who study volatiles and those who study mycotoxins do not see themselves as belonging to a single scientific community. They attend different conferences, read and publish in different journals, use different scientific approaches and often communicate using different scientific jargon. This means that the two scientific communities rarely have a chance to learn from one another. In this essay, we attempt to introduce and link the study of fungal compounds circumscribed by their volatility to the study of those fungal compounds circumscribed by their toxicity. 

## 2. What Are Toxins? Definitions and Distinctions

Toxicology is the branch of science that deals with the study of the detrimental effects of chemicals on biological systems as well as their detection and properties. When substances cause harmful effects to living systems in low doses, they are labeled “toxins” or “poisons.” In popular usage, the nouns “toxin” and “poison” often are used interchangeably. Specialists and most dictionaries, however, tend to limit the use of “toxin” to poisonous substances that are made by the metabolic activities of an organism. Poison has the broader meaning, while toxin refers only to those poisonous substances that are produced biologically. Thus, arsenic is a poison but it is not generally called a toxin. Confusingly, while the noun forms “poison” and “toxin” carry a distinction that the latter are of natural origin, the adjective forms “toxic” and “poisonous” are used interchangeably. Arsenic properly can be called a “toxic chemical” or a “toxic substance.” Similarly, the term “toxicant” is often used synonymously with poison. Finally, it should be pointed out that the word “biotoxin” defined as “a poisonous substance produced by a living organism” should be considered redundant [1,2].

The most crucial variable associated with toxic substances is the dose. The more toxic the substance is, the smaller the amount needed before it causes harm. Exposures are frequently categorized as acute *vs.* chronic. Acute toxicity is a fast response, after a brief exposure, usually at a high dose. Chronic toxicity occurs from prolonged, repeated exposures over a long period of time, often at low doses. For human beings, the major routes of exposure are through the lungs (inhalation), the mouth (dietary) and the skin (dermal). Respiratory exposure usually has the most rapid systemic action. Thus, concentrated toxicants in the volatile form are more likely to cause a rapid onset of symptoms than are toxins encountered through the diet. Many excellent textbooks are available that review basic toxicological principles and definitions [2,3,4].

Untold numbers of chemicals, both anthropogenic and biogenic, are toxic to some organism or other. When the toxicity is towards organisms that humans wish to obliterate, we often call these chemicals “drugs” and use an entirely different frame of reference for our nomenclature. For example, fungal metabolites such as penicillin that are selectively toxic to bacteria are called “antibiotics.”

For compounds toxic to humans, it is common to classify them by their mode of action as asphyxiants, narcotics, carcinogens, mutagens, teratogens, nephrotoxins, neurotoxins and the like. Another common way to classify toxins is based on their producing organism, either broadly (e.g., animal toxin) or more narrowly (e.g., dinoflagellate toxin). Some animals toxins are called “venoms” and include poisonous substances produced by snakes and stinging insects [5]. Perhaps the best-known and best-studied toxins are those produced by bacteria and include those associated with diseases such as diphtheria, botulism, and tetanus [6]. Famous plant toxins include ricin from the castor bean plant and strychnine from the Saint-Ignatius’ bean [7].

The definition of toxin has been addressed by the international legal community. After World War I, the Geneva Convention banned “asphyxiating, poisonous or other gases and bacteriological methods of warfare.” Because plant toxins such as ricin and fungal toxins such as aflatoxin are highly lethal but neither gaseous nor bacteriological, they have received special attention in subsequent international conventions concerning biological and chemical agents [8]. By definition, toxins are chemicals that are the product of biology, and depending on one’s perspective are examples of biological or chemical warfare. An authoritative definition of biological agents finally was formulated by the World Health Organization in a 1970 report that described biological agents as those that depend for their effects on multiplication within the target organism while “toxins are poisonous products of organisms.” Unlike biological agents, “toxins are inanimate and not capable of reproducing themselves.” In each case, they are intended for use in warfare to cause death or disease in targeted human, animal or plant populations. The 1972 Convention, usually referred to as the Biological Weapons Convention, came up with a list of prohibited substances, but did not give a generic definition of these substances [9,10,11]. The International Committee of the Red Cross maintains a resource center where the texts of these various agreements are available (see: http://www.icrc.org/eng/resources/index.jsp). Finally, the advent of recombinant DNA technology necessitated an extension of the concept to include toxic molecules that are constructed by biotechnology. Thus, as defined by 18 USCS § 178 a toxin is “the toxic material or product of plants, animals, microorganisms (including, but not limited to, bacteria, viruses, fungi, rickettsia or protozoa), or infectious substances, or a recombinant or synthesized molecule, whatever their origin and method of production [12].”

## 3. What Are Mycotoxins? 

Mycotoxins are an example of a category of toxins defined by the producing organism. The term “mycotoxin” entered the scientific lexicon in the early 1960s when a large die-off of turkeys (“Turkey X disease”) was linked to consumption of a poultry feed contaminated with metabolites from the mold *Aspergillus flavus*; the metabolites were later named aflatoxins [13]. Subsequently, many other toxic fungal metabolites were brought together under the ‘mycotoxin” rubric [14,15,16,17]. The traditional definition of mycotoxins as used by most authors is that they are secondary metabolites produced by microscopic fungi that are capable of causing disease and death in humans and other animals at low concentrations (see below for an expansion of this definition based on genomic data). Most human exposure to mycotoxins is through the diet, but dermal and inhalation exposures also occur. Curiously, the poisonous substances produced by mushrooms are almost never called mycotoxins; rather they are called “mushroom poisons” or “mushroom toxins [18]. Examples include amatoxin, corellanine, gyromitrin, coprine, and muscarine [19].

Like all toxicoses, the symptoms of the diseases caused by mycotoxins (“mycotoxicoses”) depend on the type and amount of mycotoxin; the length of the exposure; the age and the sex of the exposed organism; and many other parameters involving genetics, health status and interactions with other physiological factors. The severity of a mycotoxicosis can be increased by stresses like starvation and alcoholism. In turn, mycotoxicoses can heighten vulnerability to microbial diseases and worsen the effects of malnutrition [18]. The major mycotoxins associated with human and veterinary diseases include aflatoxin, citrinum, ergot alkaloids, fumonisins, ochratoxin A, patulin, and the trichothecenes [20,21,22,23]. Mycotoxins exert their toxicities with different mechanisms. For example, aflatoxins intercalate with DNA, fumonisin is an inhibitor of sphingolipid biosynthesis, and trichothecenes interfere with protein synthesis [24,25]. 

All the compounds currently classified as mycotoxins are biosynthesized by secondary metabolic processes via multistep pathways. The term “secondary metabolite” encompasses a diverse array of low molecular weight natural products that are usually produced after primary growth has stopped [26,27]. While secondary metabolites are “small molecules” when compared to macromolecules such as nucleic acids and proteins, they have higher molecular weights than VOCs. Mycotoxins are not volatile and the mycotoxins produced by growing colonies of mold do not “off gas” into the air, although dermal absorption of mycotoxins is possible [28]. 

The study of mycotoxin biosynthesis has played an important role in our understanding of the genetics of secondary metabolite biosynthesis. The aflatoxin pathway is one of the best-studied pathways of fungal secondary metabolism. In the early genomics era, the characterization of genes involved in aflatoxin biosynthesis showed that they were clustered (*i.e*., tightly linked) on the chromosome, establishing a paradigm for other secondary metabolites [29,30,31,32,33,34]. Biosynthetically, aflatoxin is a polyketide, while penicillin, a non-ribosomal polypeptide, is another secondary metabolite whose genes also were found to be clustered [35,36]. It soon became apparent that the first step in the biosynthesis of a secondary metabolite generally is catalyzed by one of five multi-domain enzymes: nonribosomal peptide synthases (NRPSs), polyketide synthases (PKSs), hybrid NRPS–PKS enzymes, prenyltransferases (DMATSs), and terpene cyclases (TCs). With the exception of terpene cyclases, which are highly variable, the genetic signature of these multi-domain enzymes can be used as a way to find secondary metabolite gene clusters in genomics data. They provide a convenient way to search fungal genome sequences for secondary metabolites genes [35,37,38]. Several web based tools have been developed which can be used to scan genomics data to speed up the identification of secondary metabolite gene clusters. One such software tool is SMURF (Secondary Metabolite Unknown Regions Finder at www.jcvi.org/smurf/) based on the hallmarks of fungal secondary metabolite biosynthetic pathways: backbone genes, gene clustering, and characteristic protein domain content [39]. A similar tool is the Antibiotics and Secondary Metabolite Analysis Shell program or AntiSMASH program [40]. In addition, there are several more specialized software tools such as CLUster Sequence Analyzer (CLUSEAN), NRPS Predictor (NRPSPredictor), ClustScan, Structure Based Sequence Analysis of Polyketide Synthetases (SBSPKS) and Natural Products searcher (NP searcher) [41]. Finally, mycotoxin and other secondary metabolite gene clusters can be identified through gene expression patterns detected by EST, microarray or RNA-Seq data. Motif Independent Detection Algorithm for Secondary Products (MIDDAS-M) was developed based on this kind of transcriptome analysis. The MIDDAS-M program has the advantage that it detects only those secondary metabolite gene clusters that are expressed [42].

In summary, the application of bioinformatics approaches to DNA sequence data for fungal genomes has generated a huge amount of data about secondary metabolite clusters, including genes for mycotoxins like aflatoxins, ochratoxins, fumonisins, gliotoxins, and sterigmatocystin. Placed in another context, it becomes apparent that an important attribute of mycotoxins beyond their physiological impact, is that they are biosynthesized in a characteristic way. We suggest a more precise definition of mycotoxins: Mycotoxins are secondary metabolites genetically encoded by clustered genes and produced by microscopic fungi that are capable of causing disease and death in humans and other animals at low concentrations.

## 4. What Are Volatile Organic Compounds (VOCs)? 

Volatile organic compounds (VOCs) are a large group of carbon-based chemicals with low molecular weights and high vapor pressure. They dissipate rapidly in terrestrial environments. Many of the best known VOCs are made by industrial activities, but numerous VOCs also are produced by living organisms as part of their metabolic processes. Industrial VOCs are nearly ubiquitous in modern society and are used in paint thinners, air fresheners, automotive products, dry cleaning fluids and the like; are generated by combustion and evaporation of petroleum-based products; and used to manufacture products like plastics, pharmaceuticals, and other major goods of modern society. Some anthropogenic VOCs are associated with air pollution and the contamination of ground water [43]. For toxigenic substances in the gas phase, inhalation exposure provides a rapid entry into the systemic circulation, causing toxic effects distant from the entry route. Dermal uptake is also an important route of exposure to volatile and semi-volatile substances. There is considerable evidence that in indoor environments transdermal uptake may occur at rates that are comparable to or larger than inhalation uptake [44,45]. 

The potential toxicity of industrial VOCs is broadly accepted and many of them are classified as hazardous or toxic by federal laws. For example, benzene is a human carcinogen while formaldehyde, perchloroethylene, and styrene are "reasonably anticipated to be human carcinogens." Several published studies have shown that exposure of several volatile organic compounds such as benzene and formaldehyde lead to adverse health effects especially hematological toxicities and exacerbation of allergy/asthma [46,47,48,49]. Industrial workers who have prolonged exposure to hazardous volatile compounds in the workplace and individuals who occupy homes exposed to emissions from heavy motor vehicle traffic are at particularly high risk of developing health problems. Short-term inhalation exposure to industrial VOCs has been associated with respiratory tract irritation, headaches, and visual impairment. Inhalation and dermal exposure to toxigenic gas phase compounds provide rapid entry into the systemic circulation; subsequent toxic effects distant from the entry route can include damage to the liver, kidneys, and central nervous system. There is a truly vast literature about the health effects and regulation of anthropogenic VOCs, especially with respect to air pollution control and the safety of indoor work and home environments. Because of their potential threat to public health, legal definitions and lists of regulated substances have been produced by several United States government agencies (see: http://toxics.usgs.gov/definitions/vocs.html). For example, the acronym BTEX is the well-known term used for benzene, toluene, ethylbenzene, and xylene-volatile aromatic compounds typically found in petroleum products such as gasoline and diesel fuel. The Agency for Toxic Substances and Diseases Registry (ATSDR) maintains comprehensive on line resources (see: http://www.atsdr.cdc.gov/). In many cases, safe exposure limits have been determined by groups such as the National Institute for Occupational Safety and Health, the Occupational Safety and Health Administration, The American Conference of Governmental industrial Hygienists, and the Environmental Protection Agency. These groups provide encyclopedic toxicological data for specific VOCs. The European Union and other international groups also have developed guidelines for their regulation. Because of the dynamic nature of the internet, URLs for these agencies and professional societies are not provided here; the reader is advised to search appropriate search terms in order to find points of access.

## 5. What Are Biogenic VOCs?

In contrast to industrial VOCs, many of which are well known toxicants, far less known about the ability of biogenic VOCs to impact human health. Hundreds of VOCs are produced by plant and microbial metabolism [50,51]. These biogenic VOCs encompass many different chemical classes including alcohols, aldehydes, ketones, amines, terpenes, aromatics, as well as halogenated and sulfur based compounds. Atmospheric chemists have shown that large quantities of non-methane organic compounds are emitted into the atmosphere from biogenic sources, mainly from vegetation. These biogenic volatiles include isoprene, monoterpenes, sesquiterpenes, and oxygenated compounds such as methanol, hexane derivatives, 2-methyl-3-buten-2-ol, and 6-methyl-5-hepten-2-one. In the troposphere, these organic compounds react with hydroxyl and nitrate radicals, and with ozone, and lead to the formation of secondary organic aerosols [52,53]. These aerosols have been studied mostly in the context of the chemistry of the lower troposphere [54] and there is growing recognition that they may contribute to air pollution [55]. Bacterial VOCs and green plant VOCs have received more attention than fungal VOCs [56,57,58]. To date, over 1700 VOCs have been described from plants [59] while only approximately 300 VOCs have been characterized from fungi, nor are most of these fungal-associated volatile compounds unique to fungal metabolism [60,61,62]. They include fatty acids and their derivatives (e.g., hydrocarbons, aliphatic alcohols and ketones), aromatic compounds, nitrogen containing compounds, and volatile sulfur compounds. All fungi emit different volatile blends. In the laboratory, individual fungal species produce a typical pattern of VOCs, which varies depending on growth conditions. The specific profile produced by each species or microbial consortium is strongly dependent on temperature, pH, moisture level, nutrients and age of the culture [63,64,65]. 

Fungal VOCs diffuse through and accumulate within indoor air. Complaints about poor air quality and associated negative health effects are often precipitated by the smell of mold [66]. The literature on the detection, identification and quantification of individual VOCs emitted by fungi is scattered [67]. One of the most comprehensive sources is a database comprising known bacterial and fungal volatiles (“mVOC”) that has been compiled at the University of Rostock, Germany [62] (see http://bioinformatics.charite.de/mvoc/#). Searches can be accessed by chemical names, chemical properties, structural similarities, by producing species, or by a combination of search terms. Also included is a “signature table” that plots the VOCs emitted by a given species against the VOCs of all the other microbial species in the database. 

Many biogenic VOCS have a low odor threshold, *i.e.*, the human nose is able to detect extremely low concentrations; perception of moldy, musty odors is a good indication that fungal growth is occurring [68]. *Aspergillus*, *Penicillium*, *Alternaria*, *Cladosporium*, *Mucor*, and *Ulocladium* are some of the most common VOC producing fungal genera found in indoor environments [69].

Surprisingly little is known about the biosynthetic origin of most fungal VOCs. The comprehensive review by Korpi *et al.* has a table summarizing what is known about the precursors to some of the most common microbial VOCs [61]. For example, compounds such as 3-methyl-1-butanol, 2-methyl-1-propanol, and styrene, arise from amino acids [70]. Several alkene and methyl ketones such as 2-butanone, 2-pentanone, and 2-undecanone, arise from fatty acids [63]. Eight carbon compounds such as 1-octen-3-ol, 3-octanol and 3-octanone are among the most common fungal VOCs. Although 1-octen-3-ol is sometimes called a secondary metabolite, it is better classified as a lipid degradation product. Both 1-octen-3-ol and a less volatile 10-oxo-acid are produced through the enzymatic oxidation and cleavage of linoleic and linolenic acids [71]. In *Pleurotus pulmonarius* two separate lipoxygenases may be involved in the production of 1-octen-3-ol and 10-oxo-acids [72]. The term “oxylipin” is often used to describe compounds derived from the oxidation of unsaturated fatty acids and includes fatty acid hydroperoxides, hydroxyl fatty acids, epoxy fatty acids, keto fatty acids, volatile aldehydes, and cyclic compounds. Volatile oxylipins are important components of many smells associated with off-odors in food [73]. Recent research suggests that some volatile oxylipins play essential roles in fungal morphogenesis and pathogenesis [74,75]. Finally, dimethyl disulfide, a compound that may be a good volatile indicator for both *Stachybotrys chartarum* and *Aspergillus versicolor*, is thought to be derived from methionine [76,77]. 

Because so little is known about the biosynthesis of microbial volatiles, they have not received much attention within the context of genome research, although Schultz and Dickschat reported a phylogenetic analysis of bacterial strains producing methyl ketones [56]. To our knowledge, no one to date has attempted to investigate phylogenetic relationships of enzymes involved in fungal volatile production. Given the wealth of sequence data available on line for fungal genomes, one obvious target for such a study would be the LOX family (linoleate: oxygen oxidoreductase, EC 1.13.11.12), which constitute a large gene family of non-heme-iron-containing fatty acid dioxygenases. These enzymes already have received some attention in the context of the phylogenetics of fungal sexual reproduction [78]. 

As a final point, it is important to remember that not all of the compounds detected in published surveys about fungal VOCs are made by biosynthetic pathways of the fungus. Many of them are merely incidental degradation products associated with the extracellular mode of fungal nutrition. Moreover, non-specific chemical reactions in the environment may convert some VOCs into other products, e.g., alcohols can be oxidized to aldehydes and further to carboxylic acids, and ketones react with hydroxyl radicals to form aldehydes [79]. Autocatalytic breakdown products of unsaturated fatty acids have been studied by food scientists because they cause rancid odors in food [80].

## 6. Are Some Fungal VOCs Toxic? 

Compared to the voluminous literature associated with the dangers of exposure to industrially produced VOCs, relatively little is known about the possible toxigenic properties of biogenic volatiles. Scientists studying toxic metabolites from fungi usually have not considered gas phase molecules. For example, the encyclopedic compendium entitled *Handbook of Toxic Fungal Metabolites* by Cole and Cox gives UV, IR and mass spectra of all major mycotoxins including aflatoxins, sterigmatocystins, versicolorins, ochratoxins, trichothecenes, cytochalasin, rubratoxins, tremorgens, toxic lactone, roquefortines, epipolythipiperiazine-3-6-diones, *Alternaria* toxins, secalonic acids, malformins, *Penicillium islandicum* toxins, sweet potato toxins, the virido group, slaframine, diplodiatoxin, roseotoxin B, and other toxins produced by *Aspergillus*, *Penicillium*, and *Fusarium*, yet does not have a single entry in the index under “volatile” [16]. You might say that scientists who study mycotoxins ignore the head space. 

To date, most of the VOCs emitted by fungi have never been tested for any physiological activity—either beneficial or toxigenic. Rather, the focus has been on their properties as flavor and aroma compounds [81] or for their use as indirect indictors of the presence of mold contamination [68]. Of those fungal VOCs that have been tested, however, a surprising number have been shown to have toxigenic properties. In an excellent review of microbial VOCs, Korpi *et al.* summarized the physical and chemical properties of 96 microbial volatiles found in buildings and then focused on the 15 most often reported fungal VOCs from moldy, water-damaged houses: 2-methyl-1-propanol, 3-methyl-1-butanol, 3-methyl-2-butanol, 2-pentanol, 3-octanol, 1-octen-3-ol, 2-octen-3-ol , 3-methylfuran, 2-hexanone, 2-heptanone, 3-octanone, 2-methylisoborneol, 2-isopropyl-3-methoxy-pyrazine, geosmin, and dimethyl disulphide [61]. Several of these compounds have extremely low odor thresholds. For example, 3-methy-1-butanol, which is described as “sour, sharp or malty” has an odor threshold of 0.010–35 ppm, while the “musty, earthy” odor of geosmin can be detected at several odors of magnitude lower at 0.0009 ppm. Dimethyl disulfide, variously described as smelling like “rotting meat” or “human feces” can be detected at even lower thresholds, at 0.00003 ppm. Given that composting often produces high concentrations of these offensive odor compounds, it is not surprising that scientists have attempted to study the health effects associated with composting facilities. In one study, it was shown that organic sulfur compounds were sufficiently high to cause toxic responses among personnel handling the compost [63]. In another study, volatile contaminants from a composting plant were associated with both odor complaints and irritation symptoms [82].

Much of the laboratory research on VOC exposure focuses on irritation. Using the mouse bioassay introduced by Alarie, a standard test for measuring irritancy of airborne chemicals was established by the American Society for Testing and Material in 1984 [83,84]. The concentration of VOC causing a 50% decrease in the respiratory rate (RD_50_) has been calculated for a number of common compounds and these data were summarized by Korpi *et al.* [61]. For example, the RD_50_ for geosmin is 29 ppm, for 1-octen-3-ol is 35 ppm, and for 3-octanol is 255 ppm [61]. Data on acute exposure to common microbial VOCs are scarce. Isolated experiments yielding lethal dose (LD_50_) and lethal concentration for single inhalation (LC_50_) data have been conducted sporadically in mouse, rabbit, guinea pigs, hamsters, or rats. Where data were available, most of these common biogenic VOCs were categorized as “slightly toxic”, having a LC_50_ in the range of 1000-10,000 ppm in rats. It was concluded that “the animal short-term and long term toxicity database is poor for most of the 15 selected substances,” but goes on to state reassuringly that the available data indicate “adverse effects from some of the selected substances have been reported only at doses far higher than what can be obtained when their main source is microbial” [61]. None of the 15 compounds have been evaluated for their carcinogenicity, although 3-methyl-2-butanone and 3-methyl-3-butanol were positive in the Ames test [85]. Using a luminesence test, SOS-inducing activity was detected for 2-methyl-1-propanol, 3-methyl-1-butanol, 3-methyl-2-butanol, 2-pentanol, 3-octanol, 1-octen-3-ol, 2-hexanone, 2-petanone, and 3-octanone [85]. Using human lung carcinoma epithelial cells, Chinese hamster fibroblasts and human peripheral blood cells, Kreja and Seidel tested 15 common volatiles. None of them caused chromosomal breaks or mutagenicity but DNA damage was detected under cytotoxic conditions, for all of the VOCs tested [86]. 

A number of fungal VOCs exhibit toxic effects on plant and plant pathogen growth. Experiments with *Arabidopsis* have shown that 1-octen-3-ol and several other VOCs cause inhibitory effects on growth of this model plant species [87,88,89]. Furthermore, plant pathologists have learned that several endophytic fungi, including *Muscodor albus*, produce VOCs that inhibit and kill plant pathogenic fungi and bacteria [90]. The VOCs of *M. albus* have been used for the control of post-harvest plant diseases caused by a variety of a fungi and bacteria in an application that has been termed “mycofumigation” [91]. Recently, using a series of genetic screens and biochemical assays, Alpha *et al.* showed that blends of VOCs from *M. albus* used in mycofumigation induced damage to DNA repair and cytotoxicity, raising questions about the possible safety of mycofumigation for agricultural workers [92]. 

The developmental stages of *Drosophila*, namely embryo, larva and adult, have been implemented in testing the toxicity of heavy metals, anesthetic gases, industrial solvents and other compounds [93,94,95]. In our laboratory, we have developed a similar *Drosophila* model specifically to assess the toxicity of fungal VOCs [96,97,98]. In our fly toxicity test, we use a double Petri dish system to expose *Drosophila* larvae to growing cultures of fungi, or alternatively, to expose adult flies in flasks to chemical standards of individual fungal volatiles [96,97]. In our assay, low concentrations of the vapor form of several C-8 compounds including 1-octen-3-ol are toxic to larvae and adult flies. Moreover, 1-octen-3-ol selectively affects dopaminergic neurons in adult *Drosophila* brain and induces Parkinson’s-like behavioral alterations in a fly model for this disease [96,99].

The toxicity of 1-octen-3-ol and other fungal VOCs has been studied using rodent models [100], cell based assays [86,101]; questionnaires [102]; direct exposure of human subjects [103], and human embryonic stems cells [104]. In the stem cells studies, volatile phase 1-octen-3-ol was 80 times more toxic than the volatile phase of toluene [104]. Uptake of dopamine was inhibited by vapor phase 1-octen-3-ol in human cell lines expressing the human plasma membrane dopamine transporter [99]. In addition, exposure of flies to 1-octen-3-ol stimulated the caspase-3 dependent apoptotic signaling pathway [98] and stimulated a nitric oxide mediated inflammatory response in nervous and respiratory tissues of *Drosophila melanogaster* [105].

Due to their low molecular weight, volatiles are easily transported through the air and in most cases, dissemination of VOCs from their point of origin leads to atmospheric dilution of the substance in question. In contrast, in enclosed environments, compounds may build up to higher levels. Toxicity is always a quantitative concept. All substances can become harmful at some dose, and even extremely potent compounds become harmless at low enough concentrations. The toxicity data on 1-octen-3-ol are of particular concern. This nearly ubiquitous fungal VOC is largely responsible for the musty odor commonly associated with mold-contaminated damp indoor spaces. Almost all toxicity tests have been conducted with the liquid, not volatile phase, of the compound. Perhaps this is why it is approved by the U.S. Food and Drug Administration (FDA) for use in foods and perfumes, and also has been approved by the Environmental Protection Agency (EPA) for insect lures. The EPA registration for 1-octen-3-ol states: “Octenol is not harmful to humans, to other non-target organisms, or to the environment. There is the potential for toxicity if ingested” [106]. We believe that further toxigenic testing is warranted. Furthermore, the recognition that 1-octen-3-ol and other naturally occurring VOCs have toxicological properties at low concentrations (0.5–3 ppm) that are only slightly higher than those encountered in nature suggests that they easily can build up to problematic levels in enclosed spaces and may possibly contribute to building related illness. 

## 7. Mycotoxin and Fungal VOC Research Overlap in the Study of Indoor Air Quality

Indoor air quality has become increasingly important in contemporary societies where people spend the vast majority of their time indoors [107]. In general, poor indoor air quality is associated with ineffective ventilation, off-gassing of building materials and contents; and/or damp and mold contaminated buildings. Poor indoor air quality has been associated with a group of nonspecific symptoms including headache, eye, nose, and throat irritation, dizziness, nausea, and difficulty concentrating [108]. People who spend a great deal of time indoors often develop this constellation of symptoms; most of the time, these symptoms are alleviated once individuals leave the problematic building [109]. In order to describe this loose diagnostic category, the terms “sick building syndrome,” “building related illness” and “damp building related illness” have been used [110,111,112,113]. There is no generally accepted clinical definition of this syndrome nor is there an adequate theory for what causes its occurrence [114]. Many international research efforts, especially in European countries, have studied the characteristics of indoor air that may have a negative impact on human health [115,116]. In a few cases, volatile chemicals such as ammonia or formaldehyde are implicated, however biological contaminants, in particular molds, are the prime suspects [117,118]. Most of the research on the presumptive association between building related illness and molds has focused on the possible role of mycotoxins [119,120,121,122,123]. However, it is questionable whether even high concentrations of spores and mycelial fragments contain sufficient mycotoxins to induce the wide array of reported symptoms [124,125,126]. Some skeptics insist that an etiological connection between mold toxins and “sick building syndrome” is untenable [127,128]. 

Although mycotoxins have received the lion’s share of the attention with respect to building related illness, fungal VOCs also have been postulated to cause or contribute to this elusive syndrome [123,129,130,131]. Given that the physiological effects of industrial solvent exposure include headaches and other neurological symptoms, it is of interest that people suffering from mold related illnesses frequently complain about neurological and neuropsychological symptoms, including headaches, difficulty concentrating, abnormal balance, slow blink reflex, diminished grip strength, and diminished visual performance [102,132,133,134,135]. In summary, fungal VOCs merit more study in the context of building related illness and may contribute to some of the reported neurological symptoms experienced by people who report mold-related ill health.

## 8. What’s in a Name? 

Both mycotoxins and fungal VOCs are natural products produced by fungi, but it is not clear that we should label toxic fungal VOCs as “mycotoxins”. Mycotoxins are all secondary metabolites, encoded by clustered genes that are easy to detect in genomic data. Only some fungal volatiles (e.g., the terpenoids) are secondary metabolites. Moreover, there already are other classes of toxic metabolites made by fungi that are not called mycotoxins. Terms like “antibiotic,” (compounds toxic to bacteria), “mushroom poison” (compounds made by mushrooms) and “phytotoxin” (compounds toxic to plants, or confusingly, made by plants [136]) are used to label certain other categories of fungal products with toxigenic properties.

Does exposure to fungal VOCs in indoor environments cause diseases in people? The answer is: “We do not know.” The group of symptoms associated with building related illness is so wide, and the health condition so loosely defined, that it is not yet considered a coherent disease label. Nor do we have the tools (e.g., biomarkers) to quantify and unite the symptoms of people who claim to have building related illness. Nevertheless, the data obtained from the *Drosophila* bioassay, especially with respect to Parkinsonian effects [96,99] are provocative and it would be prudent for the scientific community to become more aware of the fact that strong fungal odors should not be ignored.

This essay has attempted to draw attention to the potential negative health effects of fungal VOCs by asking the deliberately provocative question: “Are some fungal VOCs mycotoxins?” We recognize that the answer to this question depends on how you define mycotoxin. Creating stipulated scientific definitions is not as simple a task as one might think. The huge amount of time and attention devoted to crafting of a legal definition of toxin in the context of biological and chemical warfare helps make the point. The important take home lesson, however, is that the environmental health community should not overlook the fact that some fungal VOCs are capable of having a negative impact on the physiology of animal and plant systems, and perhaps to cause damage to humans who experience chronic exposure to mold infested environments. Unfortunately, the current toxicological data base is scanty for biogenic VOCs, even for the best studied ones. 

Genomic and phylogenetic data can clarify the way we define toxic VOCs and mycotoxins. Fungal VOCs (with the exception of terpenoids) and mycotoxins have different origins. Mycotoxins are all secondary metabolites biosynthesized by a series of enzymatic reactions encoded by genes that are linked (clustered) in the genome. Mycotoxin-encoding genes have played an important role in expanding our understanding of secondary metabolism and fungal genomics. On the other hand, the biosynthetic origin of fungal VOCs has not received much scrutiny by the scientific community and there is relatively little information available on the genetic basis of VOC biosynthesis. Furthermore, to date our catalogues of annotated fungal genes have not brought us much insight into the biosynthetic origin of fungal volatiles. Of the VOCs that have been studied, many are breakdown products of fatty acids, mediated by lipoxygenases. Others are made by simple biotransformation steps from amino acids. In some cases, we are not certain whether the VOCs we detect in profiles from growing fungi are the direct products of fungal metabolism or are merely incidental breakdown products associated with the osmotrophic nutritional strategy that characterizes the fungal kingdom. 

Advances in science often coincide with new technologies. In the future, genomic and phylogenetic studies no doubt will bring us more insight about the genetic basis of fungal VOC production. Such data will help us to separate those volatile compounds genuinely encoded by genes and those that are incidental breakdown products. However, we already know enough to say that most fungal volatiles are *not* secondary metabolites. Therefore, in summary we recommend that it would be best not to label the class of toxigenic VOCs made by fungi as mycotoxins.

## 9. Conclusions: Do We Need a New Term to Describe Toxic Volatiles?

The way we devise scientific jargon and group information into certain mental frameworks helps us discern patterns and relationships. Toxins are a category of biological products that are characterized by their negative physiological properties, namely they are poisonous. The circumscription of VOCs is based on physical state, namely they are compounds that easily evaporate from liquids, or more rarely, sublime from solids into gas phase molecules. VOC is a “big umbrella” rubric that encompasses both biogenic and anthropogenic VOCs. The volatiles that are of fungal origin are relatively understudied compared to anthropogenic, plant and bacterial VOCs but ecologists have discovered that many of them have biological activity as signal molecules and insect pheromones. The VOCs from molds and mildews are responsible for the odors we associate with damp, enclosed indoor environments and have received some attention for their possible contribution to building related illness. To date, the fungal volatiles that exhibit toxicity have been studied mostly by plant pathologists for mycofumigation or by building scientists for their possible deleterious effects on indoor air quality. 

Since words act as signifiers for the concepts that they are describing, perhaps it would be useful to coin a new term to describe volatile phase molecules of biological origin that have toxic properties. We suggest “*volatoxin*.” We define fungal *volatoxins* as volatile phase metabolites produced by fungi that can cause toxigenic effects in animals at physiological or slightly higher (2–10 X) concentrations. Enclosed built environments facilitate the buildup of VOCs to levels that are not found in nature and thereby create a milieu in which mold VOCs may become toxigenic. 

We recognize that there is certain arbitrariness to definitions. Nevertheless, advances in molecular and genetic approaches to toxicology are bringing a more in-depth understanding of the underlying mechanisms that cause toxicity and will provide new insights into the possible causal effects of biogenic VOCs in human disease. The scientific community now expects that fungal genome data will provide a useful guide to understanding the biology of an organism, but the power of genomic and other -omics technologies have not yet been brought to bear on the biosynthetic and degradative pathways that encode fungal VOCs. When the public health community recognizes that some fungal VOCs associated with the built environment are potentially toxigenic to people (*i.e*., are volatoxins) we hope that resources will be made available to bring the power of genomics and functional genomics approaches to the study of these frequently overlooked fungal metabolites. 

## References

[1] James R.C., Williams P., Burson J. (1985). General Principles of Toxicology. Industrial Toxicology: Safety and Health Applications in the Workplace.

[2] Klaassen C. (2013). Casarett & Doull's Toxicology: The Basic Science of Poisons.

[3] Hodgson E. (2010). A Textbook of Modern Toxicology.

[4] Manahan S.E. (2013). Fundamentals of Environmental and Toxicological Chemistry: Sustainable Science.

[5] White J., Meier J. (1995). Handbook of Clinical Toxicology of Animal Venoms and Poisons.

[6] Proft T. (2009). Microbial Toxins: Current Research and Future Trends.

[7] Nelson L.S., Shih R.D., Balich M.J. (2007). Handbook of Poisonous and Injurious Plants.

[8] Protocol for the Prohibition of the Use in War of Asphyxiating, Poisonous or other Gases, and the Bacteriological Methods of Warfare (Geneva Protocol 1925). https://www.icrc.org/ihl/INTRO/280?OpenDocument.

[9] United Nations (1970). Chemical and Bacteriological (Biological) Weapons and the Effects of Their Possible Use.

[10] Crone H.D. (1992). Banning Chemical Weapons. The Scientifc Background.

[11] Pringle L. (1993). Chemical and Biological Warfare: The Cruelest Weapons.

[12] US legal. http://definitions.uslegal.com/t/toxin/.

[13] Forgacs J., Carll W.T. (1962). Mycotoxicoses. Advan. Vet. Sci..

[14] Wyllie T.D., Morehouse L.G. (1977). Mycotoxic Fungi, Mycotoxins, Mycotoxicoses: An Encyclopedic Handbook. Volume I: Mycotoxic Fungi and Chemistry of Mycotoxins.

[15] Wyllie T.D., Morehouse L.G. (1978). Mycotoxic Fungi, Mycotoxins, Mycotoxicoses. An Encyclopedic Handbook. Volume II: Mycotoxicoses of Domestic and Laboratory Animals, Poultry, and Aquatic Invertebrates and Vertebrates.

[16] Cole R.J., Cox R.H. (1981). Handbook of Toxic Fungal Metabolites.

[17] Lacey J. (1985). Trichothecenes and Other Mycotoxins.

[18] Bennett J.W., Klich M. (2003). Mycotoxins. Clin. Microbiol. Rev..

[19] Benjamin D.R. (1995). Mushrooms: Poisons and Panceas. A Handbook for Naturalists, Mcologists and Physicians.

[20] Richard J.L., Thurston J.R. (1986). Diagnosis of Mycotoxicoses.

[21] Bhatnagar D., Lillehoj E.B., Arora D.K. (1992). Handbook of Applied Mycology. Volume V: Mycotoxins in Ecological Systems.

[22] Pitt J.I. (2000). Toxigenic fungi: Which are important?. Med. Mycology.

[23] Woloshuk C.P., Shim W.B. (2013). Aflatoxins, fumonisins, and trichothecenes: A convergence of knowledge. FEMS Microbiol. Rev..

[24] Eaton D.L., Groopman J.D. (1994). The Toxicology of Aflatoxins: Human Health, Veterinary, and Agricultural Significance.

[25] De Vries J.W., Trucksess M.W., Jackson L.S. (2002). Mycotoxins and Food Safety.

[26] Bennett J.W., Bentley R. (1989). What’s in a name? Microbial secondary metabolism. Adv. Appl. Microbiol..

[27] Bennett J.W. (1995). From molecular genetics and secondary metabolism to molecular metabolites and secondary genetics. Can. J. Bot..

[28] Boonen J., Malysheva S.V., Taevernier L., Diana Di Mavungu J., De Saeger S., De Spiegeleer B. (2012). Human skin penetration of selected model mycotoxins. Toxicology.

[29] Chang P.K., Cary J.W., Bhatnagar D., Cleveland T.E., Bennett J.W., Linz J.E., Woloshuk C.P., Payne G.A. (1993). Cloning of the *Aspergillus parasiticus*
*apa2* gene associated with the regulation of aflatoxin biosynthesis. Appl. Environ. Microbiol..

[30] Bennett J.W., Chang P.K., Bhatnagar D. (1997). One gene to whole pathway: the role of norsolorinic acid in aflatoxin research. Adv. Appl. Microbiol..

[31] Keller N.P., Hohn T.M. (1997). Metabolic pathway gene clusters in filamentous fungi. Fungal Genet. Biol..

[32] Payne G., Brown M.P. (1998). Genetics and physiology of aflatoxin biosynthesis. Ann. Rev. Plant Path..

[33] Yu J., Chang P.-K., Ehrlich K.C., Cary J.W., Bhatnagar D., Cleveland T.E., Payne G.A., Linz J.E., Woloshuk C.P., Bennett J.W. (2004). Clustered pathway genes in aflatoxin biosynthesis. Appl. Environ. Microbiol..

[34] Yu J., Bhatnagar D., Cleveland T.E. (2004). Completed sequence of aflatoxin pathway gene cluster in *Aspergillus parasiticus*. FEBS Lett..

[35] Keller N.P., Turner G., Bennett J.W. (2005). Fungal secondary metabolism – from biochemistry to genomics. Nat. Rev. Microbiol..

[36] Hoffmeister D., Keller N.P. (2007). Natural products of filamentous fungi: Enzymes, genes, and their regulation. Nat. Prod. Rep..

[37] Turner G., Machida M., Gomi K. (2010). Genomics and secondary metabolism in Aspergillus. Aspergillus Molecular Biology and Genomics.

[38] Andersen M.R., Nielsen J.B., Klitgaard A., Petersen L.M., Zachariasen M., Hansen T.J., Blicher L.H., Gotfredsen C.H., Larsen T.O., Nielsen K.F. (2013). Accurate prediction of secondary metabolite gene clusters in filamentous fungi. Proc. Natl. Acad. Sci. USA.

[39] Khaldi N., Seifuddin F.T., Turner G., Haft D., Nierman W.C., Wolfe K.H., Fedorova N.D. (2010). SMURF: Genomic mapping of fungal secondary metabolite clusters. Fungal Genet. Biol..

[40] Medema M.H., Blin K., Cimermancic P., de Jager V., Zakrzewski P., Fischbach M.A., Weber T., Breitling R., Takano E. (2011). AntiSMASH: Rapid identification, annotation and analysis of secondary metabolite biosynthesis gene clusters. Nucleic Acids Res..

[41] Fedorova N.D., Moktali V., Medema M.X. (2012). Bioinformatics approaches and software for detection of secondary metabolite gene clusters. Methods Mol. Biol..

[42] Umemura M., Koike H., Nagano N., Ishii T., Kawano J., Yamane N., Kozone I., Horimoto K., Shin-ya K., Asai K. (2013). MIDDAS-M: motif-independent de novo detection of secondary metabolite gene clusters through the integration of genome sequencing and transcriptome data. PLoS ONE.

[43] Hodgson E., Levi P.E. (1997). A Textbook of Modern Toxicology.

[44] Weschler C.J., Nazaroff W.W. (2012). SVOC exposure indoors: Fresh look at dermal pathways. Indoor Air.

[45] Weschler C.J., Nazaroff W.W. (2014). Dermal uptake of organic vapors commonly found in indoor air. Environ. Sci. Technol..

[46] Smith M.T. (2010). Advances in understanding benzene health effects and susceptibility. Annu. Rev. Public Health.

[47] Wilbur S., Wohlers D., Paikoff S., Keith L.S., Faroon O. (2008). ATSDR evaluation of health effects of benzene and relevance to public health. Toxicol. Ind. Health.

[48] Zhang L., Steinmaus C., Eastmond D.A., Xin X.K., Smith M.T. (2009). Formaldehyde exposure and leukemia: A new meta-analysis and potential mechanisms. Mutat. Res..

[49] Nurmatov U.B., Tagiyeva N., Semple S., Devereux G., Sheikh A. (2015). Volatile organic compounds and risk of asthma and allergy: a systematic review. Eur. Respir. Rev..

[50] Kesselmeier J., Staudt M. (1999). Biogenic volatile organic compounds (VOC): An overview on emission, physiology and ecology. J. Atmos. Chem..

[51] Hermann A. (2010). The Chemistry and Biology of Volatiles.

[52] Atkinson R., Tuazon E.D., Aschmann S.M. (2000). Atmospheric chemistry of 2-pentanone and 2-heptanone. Environ. Sci. Technol..

[53] Andreae M.O. (2009). A new look at aging aerosols. Science.

[54] Atkinson R., Arey J. (2003). Gas-phase tropospheric chemistry of biogenic volatile organic compounds: A review. Atmos. Environ..

[55] Carlton A.G., Pinder R.W., Bhave P.V., Pouliot G.A. (2010). To what extent can biogenic secondary organic aerosols (SOA) be controlled?. Environ. Sci. Technol..

[56] Schulz S., Dickschat J.S. (2007). Bacterial volatiles: the smell of small organisms. Nat. Prod. Rep..

[57] Piechulla B., Degenhardt J. (2014). The emerging importance of microbial volatile organic compounds. Plant Cell Environ..

[58] Kanchiswamy C.N., Mainoy M., Maffie M.E. (2015). Chemical diversity of microbial volatiles and their potential for plant growth and productivity. Front. Plant Sci..

[59] Dudavera N., Pichersky E. (2006). The Biology of Floral Scent.

[60] Chiron N., Michelot D. (2005). Mushrooms odors, chemistry and role in the biotic interactions—A review. Cryptogam. Mycol..

[61] Korpi A., Jarnberg J., Pasanen A.L. (2009). Microbial volatile organic compounds. Crit. Rev. Toxicol..

[62] Lemfack M.C., Nickel J., Dunkel M., Preissner R., Piechulla B. (2014). VOC: A database of microbial volatiles. Nucleic Acids Res..

[63] Wilkins K., Larsen K. (1995). Identification of volatile (micro) biological compounds from household waste and building materials by thermal desorption-Capillary gas chromatography-mass spectroscopy. J. High Res. Chrom..

[64] Bennett J.W., Hung R., Lee S., Padhi S., Hock B. (2013). Fungal and bacterial volatile organic compounds; an overview and their role as ecological signaling agents. The Mycota IX Fungal Interactions.

[65] Morath S., Hung R., Bennett J.W. (2012). Fungal volatile organic compounds: A review with emphasis on their biotechnological potential. Fungal Biol. Rev..

[66] Horner E.W., Miller D.J. (2003). Microbial volatile organic compounds with emphasis on those arising from filamentous fungal contaminants of buildings. ASHRAE Transact..

[67] Hung R., Lee S., Bennett J.W. (2015). Fungal volatile organic compounds and their role in ecosystems. Appl. Microbiol. Biotechnol..

[68] Magan N., Evans P. (2000). Volatiles as an indicator of fungal activity and differentiation between species, and the potential use of electronic nose technology for early detection of grain spoilage. J. Stored Prod. Res..

[69] Claeson A. (2006). Volatile organic compounds from microorganisms—Identification and health effects. Ph.D. Thesis.

[70] Bjurman J., Salhammer T. (1999). Release of MVOCs from microorganisms. Organic Indoor Air Pollutants: Occurrence, Measurement, Evaluation.

[71] Wurzenberger M., Grosch W. (1984). Stereochemistry of the cleavage of the 10-hydroperoxide isomer of linoleic acid to 1-octen-3-ol by a hydroperoxide lyase from mushrooms (*Psalliota bispora*). Biochem. Biophys. Acta.

[72] Assaf S., Hadar Y., Dosoretz C.G. (1997). 1-Octen-3-ol and 13-hydroperoxylinoleate are products of distinct pathways in the oxidative breakdown of linoleic acid by *Pleurotus pulmonarius*. Enzyme Microb. Technol..

[73] Min D.B., Smouse T.H., Chang S.S. (1989). Flavor Chemistry of Lipid Foods.

[74] Chen H., Fink G.R. (2006). Feedback control of morphogenesis in fungi by aromatic alcohols. Genes Dev..

[75] Tsitsigiannis D.I., Keller N.P. (2006). Oxylipins act as determinants of natural product biosynthesis and seed colonization in *Aspergillus nidulans*. Mol. Microbiol..

[76] Wessen B., Strom G., Palmgren U., Shoeps K.O., Nilsson M., Flanagan B., Samson R.A., Mller J.D. (2001). Analysis of microbial volatile organic compounds. Microorganisms in Home and Indoor Work Environments.

[77] Mason S., Cortes D., Horner W.E. (2010). Detection of gaseous effluents and by-products of fungal growth that affect environments. HVAC&R.

[78] Wadman M.K., de Vries R.P., Kalkhove S.I.C., Veldink G.A., Vliegenthart J.F.G. (2009). Characterization of oxylipins and dioxygenase genes in the asexual fungus *Aspergillus niger*. BMC Microbiol..

[79] Atkinson R. (2000). Atmospheric chemistry of VOCs and NOx. Atmos. Environ..

[80] Chan H.W.-S. (1987). Autoxidation of Unsaturated Lipids.

[81] Fraatz M.A., Zorn H., Hofrichter M. (2010). Fungal Flavours. The Mycota X: Industial Applications. 2.

[82] Muller T., Thissen R., Braun S., Dott W., Fischer G. (2004). (M)VOC and composting facilities. Part 2. (M)VOC dispersal in the environment. Environ. Sci. Pollut. Res. Int..

[83] Alarie Y. (1996). Irritating properties of airborne materials to the upper respiratory tract. Arch. Environ. Health.

[84] American Society for Testing and Materials (1984). Standard test method for estimating sensory irritancy of airborne chemicals. Annual Book of ASTM Standards.

[85] Nakajima D., Ishii R., Kageyama S., Onji Y., Mneki S., Morooka N., Takatori K., Goto S. (2006). Genotoxicty of microbial volatile organic compounds. J. Health Sci..

[86] Kreja L., Seidel H.J. (2002). Evaluation of the genotoxic potential of some microbial volatile organic compounds (MVOC) with the comet assay, the micronucleus assay and the HPRT gene mutation assay. Mutat. Res..

[87] Splivallo R., Novero M., Bertea C., Bossi S., Bonfante P. (2007). Truffle volatiles inhibit growth and induce an oxidative burst in *Arabidopsis thaliana*. New Pathologist.

[88] Hung R., Lee S., Bennett J.W. (2014). The effects of low concentrations of the enantiomers of mushroom alcohol (1-octen-3-ol) on *Arabidopsis thaliana*. Mycology.

[89] Lee S., Hung R., Schink A., Mauro J., Bennett J. (2014). *Arabidopsis thaliana* for testing the phytotoxicity of volatile organic compounds. Plant Growth Regul..

[90] Strobel G.A., Dirkse E., Sears J., Markworth C. (2001). Volatile antimicrobials from *Muscodor albus* a novel endophytic fungus. Microbiology.

[91] Stinson M., Ezra D., Hess W.M., Sears J., Strobel G. (2003). An endophytic *Gliocladium* sp. of *Eucryphia cordifolia* producing selective volatile antimicrobial compounds. Plant Sci..

[92] Alpha C.J., Campos M., Jacobs-Wagner C., Strobel S.A. (2015). Mycofumigation by the volatile organic compound producing fungus *Muscodor albus* induces bacterial cell death through DNA damage. Appl. Environ. Microbiol..

[93] Campbell D.B., Nash H.A. (1994). Use of *Drosophila* mutants to distinguish among volatile general anesthetics. Proc. Natl. Acad. Sci. USA.

[94] Wasserkort R., Koller T. (1997). Screening toxic effects of volatile organic compounds using *Drosophila melanogaster*. J. Appl. Toxicol..

[95] Rand M.D. (2009). Drosophotoxicology: The growing potential for *Drosophila* in neurotoxicology. Neurotoxicol. Teratol..

[96] Inamdar A.A., Masurekar P., Bennett J.W. (2010). Neurotoxicity of fungal volatile organic compounds in *Drosophila melanogaster*. Toxicol. Sci..

[97] Inamdar A.A., Zaman T., Morath S.U., Pu D.C., Bennett J.W. (2014). *Drosophila melanogaster* as a model to characterize fungal volatile organic compounds. Environ. Toxicol..

[98] Inamdar A.A., Masurekar P., Hossain M., Richardson J.R., Bennett J.W. (2014). Signaling pathways involved in 1-octen-3-ol-mediated neurotoxicity in *Drosophila melanogaster*: Implication in Parkinson’s disease. Neurotox. Res..

[99] Inamdar A.A., Hossain M.M., Bernstein A.I., Miller G.W., Richardson J.R., Bennett J.W. (2013). The fungal derived semiochemical 1-octen-3-ol disrupts dopamine packaging and causes neurodegeneration. Proc. Natl. Acad. Sci. USA.

[100] Haynes R.L., Szweda L., Pickin K., Welker M.E., Townsend A.J. (2000). Structure-activity relationships for growth inhibition and induction of apoptosis by 4-hydroxy-2-nonenal in rat 264.7 cells. Mol. Pharmacol..

[101] Kreja L., Seidel H. (2002). On the cytotoxicity of some microbial volatile organic compounds as studied in the human lung cell line A549. Chemosphere.

[102] Kilburn K.H. (2009). Neurobehavioral and pulmonary impairment in 105 adults with indoor exposure to molds compared to 100 exposed to chemicals. Toxicol. Ind. Health.

[103] Walinder R., Ernstgard L., Norback D., Wieslander G., Johanson G. (2008). Acute effects of 1-octen-3-ol, a microbial volatile organic compound (MVOC)—An experimental study. Toxicol. Lett..

[104] Inamdar A.A., Moore J.C., Cohen R.I., Bennett J.W. (2012). A model to evaluate the cytotoxicity of the fungal volatile organic compound 1-octen-3-o1 in human embryonic stem cells. Mycopathologia.

[105] Inamdar A.A., Bennett J.W. (2014). A common fungal volatile organic compound induces a nitric oxide mediated inflammatory response in *Drosophila melanogaster*. Sci. Rep..

[106] (2015). EPA—Environmental Protection Agency. http://www.epa.gov/opp00001/chem_search/reg_actions/registration/fs_G-126_05-Jul-07.pdf.

[107] Klepeis N.E., Nelson W.C., Ott W.R., Robinson J.P., Tsang A.M., Switzer P., Behar J.V., Hern S.C., Engelmann W.H. (2001). The National Human Activity Pattern Survey (NHAPS): A resource for assessing exposure to environmental pollutants. J. Expo. Anal. Environ. Epidemiol..

[108] Environmental Protection Agency. http://www.epa.gov/iaq/pdfs/sick_building_factsheet.pdf.

[109] Polizzi V., Adams A., Picco A., Adriaens E., Lenoir J., Peteghem C., Saeger S., Kimpe N. (2011). Influence of environmental conditions on production of volatiles by *Trichoderma atroviride* in relation with the sick building syndrome. Build. Environ..

[110] Baechler M.D., Hadley D.L., Marseille T.J., Berry M.A. (1991). Sick Building Syndrome. Sources, Health Effects, Mitigation.

[111] Burge H.A. (1996). Health effects of biological contaminants. Indoor Air and Human Health.

[112] Straus D.C. (2004). Sick building syndrome. Advances in Applied Microbiology.

[113] Pestka J.J., Yike I., Dearborn D.G., Ward M.D., Harkema J.R. (2008). *Stachybotrys chartarum*, trichothecene mycotoxins, and damp building-related illness: New insights into a public health enigma. Toxicol. Sci..

[114] Redlich C.A., Sparer J., Cullen M.R. (1997). Sick-building syndrome. The Lancet.

[115] Institute of Medicine (US) Committee on Damp Indoor Spaces and Health (2004). Damp Indoor Spaces and Health.

[116] Heseltine E., Rosen J. (2009). World Health Organization Guidelines for Indoor Air Quality: Dampness and Mould.

[117] Li D.W., Yang C.S. (2004). Fungal contamination as a major contributor to sick building syndrome. Adv. Appl. Microbiol..

[118] Bush R.K., Portnoy J.M., Saxon A., Terr A.I., Wood R.A. (2006). The medical effects of mold exposure. J. Allergy Clin. Immunol..

[119] Dales R.E., Burnett R., Zwanenburg H. (1991). Adverse health effects among adults exposed to home dampness and molds. Am. Rev. Respir. Dis..

[120] Hendry K.M., Cole E.C. (1993). A review of mycotoxins in indoor air. J. Toxic. Environ. Health.

[121] Yang C., Johanning E., Hurst C., Crawford R.L., Garland J.L., Lipson D.A., Mills A.L., Stetzenback L.D. (1996). Airborne fungi and mycotoxins. Manual of Environmental Microbiology.

[122] Robbins C.A., Swenson L.J., Nealley M.L., Kelman B.J., Gots R.E. (2000). Health effects of mycotoxins in indoor air: a critical review. Appl. Occup. Environ. Hyg..

[123] Straus D.C. (2009). Molds, mycotoxins and sick building syndrome. Toxicol. Ind. Health.

[124] Peraica M., Radic B., Luci A., Pavlovic M. (1999). Toxic effects of mycotoxins in humans. Bull. World Health Organ..

[125] Rao C.Y., Burge H.A., Brain J.D. (2000). The time course of response to intratracheally instilled toxic *Stachybotrys chartarum* spores in rats. Mycopathologia.

[126] Kuhn D.M., Ghannoum M.A. (2003). Indoor mold, toxigenic fungi, and *Stachybotrys chartarum*: infectious disease perspective. Clin. Microbiol. Rev..

[127] Chapman J.A., Terr A.I., Jacobs L., Charlesworth E.N., Bardana E.J. (2003). Toxic mold: phantom risk *vs.* science. Ann. Allergy Asthma Immunol..

[128] Hardin B.D., Kelman B.J., Saxon A. (2003). Adverse human health effects associated with molds in the indoor environment. J. Occup. Environ. Med..

[129] Mølhave L. (1992). Controlled experiments for studies of the sick building syndrome. Ann. N. Y. Acad. Sci..

[130] Mølhave L., Liu Z., Jørgensen A.H., Pedersen O.F., Kjægaard S.K. (1993). Sensory and physiological effects on humans of combined exposures to air temperatures and volatile organic compounds. Indoor Air.

[131] Mølhave L., Lippmann M. (2009). Volatile organic compounds and the sick building syndrome. Environmental Toxicants: Human Exposures and their Health Effects.

[132] Gray M.R., Thrasher J.C., Crago R., Madison R.A., Arnold L., Campbell A.W., Vojdani A. (2003). Mixed mold mycotoxicoses: immunological changes in humans following exposure in water-damaged buildings. Arch. Environ. Health.

[133] Empting L.D. (2009). Neurologic and neuropsychiatric syndrome features of mold and mycotoxin exposure. Toxicol. Ind. Health.

[134] Kilburn K.H. (2002). Inhalation of molds and mycotoxins. Eur. J. Oncol..

[135] Kilburn K.H. (2004). Role of molds and mycotoxins in being sick in buildings: Neurobehavioral and pulmonary impairment. Adv. Appl. Microbiol..

[136] Graniti A., Wood R.K., Ballio A., Graniti A. (1972). The evolution of the toxic concept in plant pathology. Phytotoxins in Plant Diseases.

